# Appearance Reveals Music Preferences

**DOI:** 10.1177/01461672211048291

**Published:** 2021-09-28

**Authors:** Laura Tian, Ravin Alaei, Nicholas O. Rule

**Affiliations:** 1 University of Toronto, Ontario, Canada

**Keywords:** accuracy, face perception, music, preferences, person perception

## Abstract

Disclosing idiosyncratic preferences can help to broker new social interactions. For instance, strangers exchange music preferences to signal their identities, values, and preferences. Recognizing that people’s physical appearances guide their decisions about social engagement, we examined whether cues to people’s music preferences in their physical appearance and expressive poses help to guide social interaction. We found that perceivers could detect targets’ music preferences from photos of their bodies, heads, faces, eyes, and mouths (but not hair) and that the targets’ apparent traits (e.g., submissiveness, neatness) undergirded these judgments. Perceivers also desired to meet individuals who appeared to match their music preferences versus those who did not. Music preferences therefore seem to manifest in appearance, regulating interest in others and suggesting that one’s identity redundantly emerges across different types of cues. People may thus infer others’ music preferences to identify candidates for social bonding.

Individuals’ preferences disclose much about them. For instance, one’s Facebook likes, room decor, dress, and favorite movies, books, and songs reveal their values, personalities, and social traits (Gosling et al., 2002; Kosinski et al., 2013; Rentfrow, Goldberg, & Zilca, 2011). Music may rank as especially important among idiosyncratic preferences, often acting as “badges of identity” and seeds for “taste cultures,” or social groups defined by shared preferences across multiple domains (North & Hargreaves, 1999; Rentfrow, 2012; Rentfrow & Gosling, 2007). Accordingly, new acquaintances discuss shared music preferences more than any other conversational topic (Rentfrow & Gosling, 2006), perhaps recognizing the value of music preferences in providing a window into important social values and identities, such as political orientation and religiosity (Boer et al., 2011).

But how do people spot those who may share their interests and, more specifically, their music preferences? Research has shown that aspects of people’s appearances, such as their dress, grooming, expressions and mannerisms, and even their static facial features often (deliberately and incidentally) reveal their traits, values, and identities (e.g., Ekman et al., 1969; Naumann et al., 2009). Yet, few empirical studies have examined how people judge others based on inferences of tastes (cf. Rentfrow & Gosling, 2003) and none have investigated how appearance may reflect music preferences. Given the importance of music preferences in communicating identity, we investigated whether people display and detect music preferences using appearance information. By inferring others’ music preferences quickly from their appearance (and by communicating one’s own), people might efficiently exchange valuable social information, which may then inform social behavior (e.g., approach vs. avoid; Rentfrow, Goldberg, & Zilca, 2011).

## What Do Music Preferences Communicate?

People gravitate toward music that reflects their values, habits, personality traits, and sexual development (e.g., North & Hargreaves, 1999; Primack et al., 2009; Rentfrow & Gosling, 2007). For instance, people who enjoy electronica, pop, and rap are more likely to be extraverted, and people who like heavy metal and punk tend to be more impulsive, risk-seeking, and exhibit higher levels of delinquency, Machiavellianism, and openness to new experiences than fans of “lighter” genres (e.g., pop; Boer et al., 2011; Rentfrow & Gosling, 2007; Swami et al., 2013).

People who enjoy classical music and opera are also more open to new experiences, but obtain better grades, more advanced degrees, and higher status romantic partners (DiMaggio, 1982; Roe, 1992). Such findings suggest that music preferences not only relate to fundamental traits but also predict important life outcomes and help people to express, form, and maintain their individual identities.

Beyond individual differences, people’s music preferences also stem from and reflect their social identity (e.g., Lonsdale & North, 2009). For instance, observers might label people standing near rock music venues clad in leather jackets, ripped jeans, and tattoos as “metal heads.” Similarly, “ravers” often pack into electronic dance halls dressed in tie-dye clothes and beaded accessories, whereas posh evening couture and black tie are common during opening nights at the symphony. These subcultures demonstrate how music preferences can bind individuals together to form social groups and create social identities (Boer et al., 2011; Gardikiotis & Baltzis, 2011). Similar to other social identities, members of music subcultures favor people who share their music preferences, young children prefer other children who like and know the same songs as them, and music fans guard against outsiders whose interest in their music appears insincere (Lonsdale & North, 2009; Soley & Spelke, 2016; see also Tarrant et al., 2001). Such favoritism or homophily also appears in other domains (e.g., friend groups, fraternities, and sports teams) in which members share similar interests and opinions, validating one’s own perspective and experiences in ways that improve self-esteem and well-being (Hehman et al., 2018; Lonsdale, 2020).

Indeed, the role of music in forming social bonds and identities is well-documented and ubiquitous across cultures and histories. One theory posits that human musicality (i.e., biological mechanisms underlying music production and appreciation) has been evolutionarily advantageous because music facilitated efficient social bonding, which permitted human groups to grow in size and complexity (Savage et al., 2020). Unlike other forms of bonding (e.g., grooming, play), music production and appreciation allow people to concurrently bond with large groups and, importantly, implicitly signal shared social and cultural knowledge. To illustrate, music artists like Pete Seeger and Bob Dylan played key roles in developing and proliferating counterculture ideologies prevalent during the 1960s to 1970s, and political music, such as national anthems and protest songs, inspires patriotism and mobilizes activists (Eyerman, 2002; Futrell et al., 2006). Moreover, music often amplifies and coheres social movements: Songs’ lyrical themes and stylistic patterns have reflected the experiences of (often marginalized) social groups throughout history, thereby helping people to develop, express, and reinforce their social identity (Martiniello & Lafleur, 2008; Pettijohn & Sacco, 2009). People’s music preferences can therefore supply clues to central social identities, such as people’s age, race, gender, nationality, social class, and religiosity when such information is ambiguous and unavailable (and vice versa; for example, Dixon, 1981; Larson, 1995).

Given that music preferences coincide with individual and social identities, people may display and infer music preferences from appearance for quick identification. Specifically, appearance stereotypes related to both individual traits (e.g., ostensible extraversion, neatness) and social identities (e.g., age, race, gender) may validly signal music preferences and these perceptions may then act as social bridges that help people identify new social opportunities.

## Inferring Music Preferences

Judgments of others’ music preferences would likely rely on stereotypes about how other fans of those particular music styles behave and look (Lonsdale & North, 2011). These stereotypes include ideas about how fans of different genres dress, their traits and demographics (including self-esteem, delinquency, social class, age, gender, and education), and their other interests (e.g., Griffiths, 2010; Lonsdale & North, 2011). Lena and Peterson (2008), for example, noted that music genres related to distinct dress styles: eccentric dress with avant-garde genres, genre-stereotypic dress with scene-based genres (e.g., punk), mainstream or mass-marketed dress with industry-based genres (e.g., popular music), and muted and stereotypic dress with traditionalist genres (e.g., bluegrass). Moreover, both laypeople and experts have expectations of “appropriate dress” for different music genres (Griffiths, 2010). People will use these stereotypes to judge others’ music preferences from their appearance, with their accuracy hinging on the extent to which the stereotypes contain kernels of truth (see Alaei & Rule, 2016).

Beyond dress, however, people may also rely on more static aspects of how others look, such as their facial appearance, which plays an important role in person perception more generally (e.g., Perrett, 2010; Zebrowitz, 1997). Although one might expect that judgments of others’ music preferences primarily rely on a person’s deliberate style of dress (i.e., body-related cues; Wapnick et al., 1998), decades of research in social perception support the face’s central role in a variety of social judgments and decisions (e.g., Perrett, 2010; Zebrowitz, 1997).

Specifically, individuals join groups and befriend others who share similar facial features and, conversely, people also *perceive* group members with similar facial features as more psychologically homogeneous (Dasgupta et al., 1999; Hehman et al., 2018). Similar findings suggest that facial cues can divulge ambiguous group memberships, such as nationality, linguistic identity, religiosity, and sexual and political orientation (see Tskhay & Rule, 2013, for a review). Perceivable intergroup differences in fundamental traits, such as attractiveness, dominance, trustworthiness, and warmth can explain these judgments. And, imaginably, such differences may also reveal music preferences. For instance, stereotypes suggest that people who prefer upbeat “pop” music may appear more conventional and enthusiastic (with mainstream dress styles and positive facial expressions) than people who prefer “rebellious” genres, such as rap (Rentfrow & Gosling, 2003).

Notably, previous research demonstrates that these traits may also become expressed in music preferences through an underlying structure of dimensions that represent clusters of genres (Rentfrow, Goldberg, & Levitin, 2011; Rentfrow & Gosling, 2003). Researchers have found similar dimensions of music preferences across diverse samples (e.g., Gardikiotis & Baltzis, 2011; Nowack, 2018), suggesting that fundamental dimensions organize people’s music preferences. We therefore examined whether people’s appearance reflects their preferences for music dimensions.

## Current Work

Because identity is expressed through appearance and music preferences form part of identity, we hypothesized that people’s appearance would provide cues to their music preferences that others might then use to decide whether to socially engage with them. We had no hypotheses about the exact appearance components that may reveal music preferences but instead utilized a data-driven approach that gradually isolated the features underlying social trait judgments and, by extension, music preference judgments.

**Figure 1.**
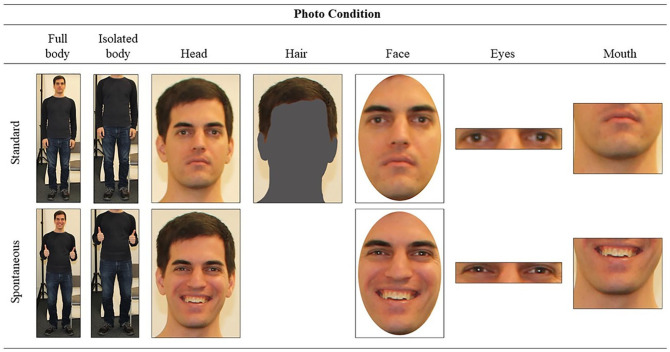
Example stimuli across photo conditions. *Note.* This target reported enjoying reflective/complex music the most and enjoying upbeat/conventional music the least.

Specifically, we began by investigating whether people could detect people’s self-reported music preferences from standardized and spontaneously posed full body photos in Study 1. In addition to using standardized photos (the standard for experimental control), we also examined spontaneous photos because they sometimes reveal more identity information (Naumann et al., 2009). After results showed that people could detect music preferences from standardized and spontaneous full body photos, we then examined separate components of standardized full body photos to uncover which components of targets’ appearance revealed their music preferences. The final selection included isolated bodies, heads, hair, faces, eyes, and mouths (see Figure 1). Past person perception research has implicated these components in myriad social trait judgments: Whereas isolated bodies and hair provide clues to grooming habits that often reflect subcultural differences (Lena & Peterson, 2008), facial feature configuration cues (such as facial width-to-height ratios or babyfacedness; Berry & McArthur, 1985; Carré et al., 2009) can influence perceptions of traits, such as aggression or submissiveness. And even isolated facial features, such as pupil dilation, eye gaze, and mouth width can reveal fear, intelligence, and leadership potential (Adams & Kleck, 2005; Re & Rule, 2016; van der Wel & van Steenbergen, 2018). Importantly, these appearance-based trait judgments often validly predict actual personality traits and group memberships, which also coincide with music preferences (e.g., Rentfrow & Gosling, 2006).

Upon determining that participants could detect targets’ music preferences from their appearance in Study 1, we examined whether impressions of the targets’ traits (such as perceived attractiveness, dominance, distinctiveness, energy, and neatness) explained these accurate judgments in Study 2. Finally, we explored how music preference judgments might affect social intentions in Study 3, thus providing empirical evidence that inferences of music preferences facilitate social opportunities. We report all data exclusions, manipulations, and measures for all studies.

## Study 1: Detection

In Study 1, we investigated whether first impressions of targets’ music preferences from their appearance (in standardized and spontaneous poses) matched their self-reported actual music preferences. Notably, targets arrived at the lab naïve about our request to photograph them and measure their music preferences, which allowed us to test whether people’s daily, incidental appearance reflects their music preferences. Importantly, we digitally blurred any explicit music preference cues in clothing (e.g., a band logo) and statistically adjusted for targets’ reported age, race, and gender (see Marshall & Naumann, 2018). We used photos of various body parts (including the full body, isolated body, head, hair, face, eyes, and mouth) to determine where music preference cues may lie.

Analysis focused on the fundamental dimensions that organize people’s music preferences. Specifically, various samples demonstrate a similar structure to music preferences in which genres cluster into reliable factors (see Rentfrow, 2012, for review). We follow those identified in Rentfrow and Gosling’s (2003) original analyses: *energetic/rhythmic* (electronica/dance, rap, and soul/funk), *intense/rebellious* (alternative, heavy metal, and rock), *reflective/complex* (blues, classical, folk, and jazz), and *upbeat/conventional* (country, pop, religious, and soundtracks).

### Method

#### Targets

We recruited 289 Canadian undergraduate students to serve as targets (206 women, 82 men, one “other”; *M*_age_ = 20.34 years, *SD* = 3.90; 46.37% East Asian, 21.11% White, 15.92% South Asian, 11.53% “Other,” 2.08% Black, and 2.08% Hispanic; *M*_years in Canada_ = 11.93 years, *SD* = 8.76). We planned the sample size to afford at least 95% power (final power: 97%) in a multivariate regression model with four predictors when assuming the average effect size in comparable interpersonal perception studies (*r*_ES_ = .29; Tskhay & Rule, 2013). We instructed targets to first pose with their hands by their sides and a neutral expression on their faces (*standardized* pose photos), and then in any way they wished (*spontaneous* pose photos). Later in the experimental session, each target completed the Short Test of Music Preferences (STOMP; Rentfrow & Gosling, 2003),^1^ which asks one’s preference for 14 common music genres (alternative, blues, classical, country, electronica/dance, folk, heavy metal, jazz, pop, rap/hip-hop, religious, rock, soul/funk, and soundtracks; 1 = *not at all*, 7 = *a great deal*). We also assessed targets’ preferences for happy and sad music for exploratory analyses not reported here. See Supplemental Table S5 for zero-order correlations.

We used Adobe Photoshop CS6 to crop and standardize stimulus photos by height before presenting them to perceivers, keeping images of isolated features (bodies, eyes, etc.) in their original size and image resolution. The full body photos consisted of the head and body, which we cropped to show only the targets’ bodies for the isolated body condition. We subsequently isolated targets’ hair,^2^ eyes, mouth, and face (elliptically cropping the head photos to show only the faces without hair, face shape, or neck) because each has provided valid cues to identity and preferences in previous work (e.g., Brown & Perrett, 1993; Tskhay & Rule, 2013).

#### Perceivers

We recruited 3,953 participants from Amazon’s Mechanical Turk (MTurk) across seven sequential studies (ensuring that participants could not participate in more than one), each asking participants to judge targets from one condition (*M* = 564.71 participants per condition, *SD* = 4.50; that is, standardized or spontaneous full bodies, isolated bodies, heads, hair, faces, eyes, or mouths; see Table 1 for demographic information)^3^ and one pose condition without time restrictions. Each participant rated a random subset of 21 targets’ preferences for the musical genres described above so that approximately 20 perceivers rated each target for each photo condition using the same scale points.^4^ Ratings showed acceptable interrater reliability or better (see Supplemental Table S7).

**Table 1.** Summary Statistics for Perceivers by Photo Condition in Study 1.

Study 1 perceivers						
Photo condition	*n*female	*n*male	*n*other	*M*age (years) (*SD*)	*M*raters per target (*SD*)	Race
Full body	360	199	1	36.99 (12.96)	19.22 (3.55)	77.64% White, 9.66% Latin/Hispanic, 7.33% Black, 2.50% Other, 1.61% East Asian, 1.25% South Asian
Isolated body	365	198	1	38.94 (13.23)	20.12 (1.69)	79.79% White, 6.98% Black, 3.94% Latin/Hispanic, 3.40% East Asian, 2.86% Other, 2.86% South Asian
Head	363	194	2	39.13 (13.75)	20.75 (1.32)	73.89% White, 10.91% Black, 5.55% Latin/Hispanic, 4.29% East Asian, 3.40% Other, 1.97% South Asian
Hair	344	219	0	36.74 (11.92)	41.35 (2.68)	74.42% White, 8.77% Black, 7.16% Latin/Hispanic, 3.92% East Asian, 3.40% Other, 2.33% South Asian
Internal face	358	205	4	37.83 (13.18)	21.36 (1.62)	70.30% White, 9.30% Latin/Hispanic, 8.41% Black, 5.37% East Asian, 3.58% Other, 3.04% South Asian
Eyes	355	214	0	39.92 (13.35)	20.32 (1.80)	74.78% White, 10.02% Black, 5.72% Latin/Hispanic, 4.47% Other, 3.76% East Asian, 1.25% South Asian
Mouth	350	220	1	37.62 (12.48)	20.43 (1.88)	72.63% White, 10.38% Black, 6.08% Latin/Hispanic, 4.11% East Asian, 4.11% Other, 3.94% South Asian

*Note.* Hair photos identical across standardized and spontaneous conditions.

### Results and Discussion

A confirmatory factor analysis (CFA) conducted with R employing a WLSMV (mean- and variance-adjusted weighted least squares) estimation method (appropriate for ordinal data; Li, 2016) and listwise deletion for missing values suggested that the original STOMP dimensions showed adequate fit, χ^2^(71) = 201.92, *p* < .001, comparative fit index (CFI) = .90, root mean square error of approximation (RMSEA) = .08, 90% confidence interval [CI] = [.07, .09], goodness-of-fit index GFI = .96, adjusted goodness-of-fit index (AGFI) = .94, according to Steiger’s (1998) RMSEA (< .10) recommendations but a poor fit according to Hu and Bentler’s (1999) CFI recommendations (CFI ≥ .95; see Supplemental Figure S1 for factor loadings and covariances and Supplemental Table S6 for a variance-covariance matrix). Conducting an exploratory factor analysis to respecify the model showed an alternative but similar four-factor solution comprised of *Contemporary/Rhythmic* (electronica/dance, pop, rap/hip-hop, soundtracks), *Intense/Rebellious* (alternative, heavy metal, rock), *Reflective/Complex* (blues, classical, jazz, soul/funk), *and Unpretentious/Conventional* (country, folk, and religious) dimensions, which we report in the Supplemental Analyses.

**Figure 2.**
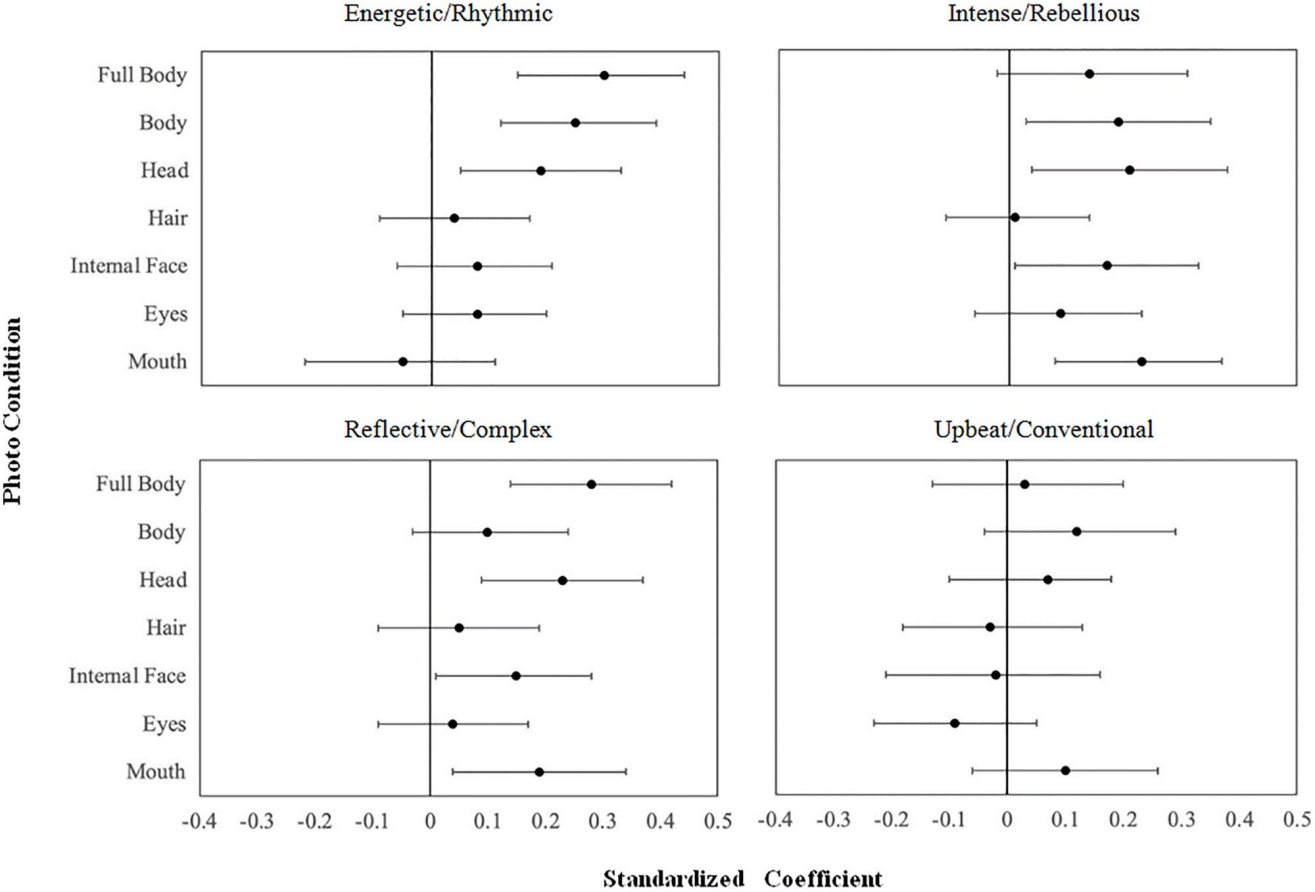
Standardized regression coefficients and confidence intervals for targets’ actual music preference regressed on overall perceived music dimension preference in Study 1. *Note.* Coefficients based on perceived music dimension preference ratings averaged across standardized and spontaneous poses, adjusted for target age, race, and gender. Error bars represent 95% confidence intervals.

We aggregated the perceivers’ mean ratings of the 14 genres into Rentfrow and Gosling’s (2003) four music dimensions (i.e., energetic/rhythmic, intense/rebellious, reflective/complex, and upbeat/conventional) and did the same for the targets’ self-reported preferences for these dimensions based on their responses to the STOMP questionnaire (see Supplemental Tables S17–S19 for zero-order correlations and descriptive statistics).^5^ We then submitted these two sets of scores to a multivariate regression in which the targets’ perceived music preference dimensions, age, race, and gender predicted their actual music preferences, estimating separate models for the standardized and spontaneous photos (see Figure 2 and Supplemental Table S8 for accuracy coefficients; see Supplemental Tables S10–S16 and S20, for the full results from each model). We statistically adjusted for the targets’ age, race, and gender because they pervasively correlated with judgments of targets’ music preferences (see Supplemental Table S9); note that a multivariate regression mitigates the family-wise error rate associated with multiple tests (Leary & Altmaier, 1980). We coded race as White versus non-White because the perceiver samples consisted of predominantly White participants (Table 1) and same-race targets thus served as the reference group (Hegarty, 2017; unfortunately, modeling all racial groups would excessively stratify and underpower these analyses).

#### Full and isolated body

Regardless of pose, targets’ perceived music preferences significantly predicted their actual music preferences for energetic/rhythmic music and reflective/complex music in the full body photos. Individuals therefore seem to express some of their music preferences in their appearance. Repeating the analysis for the isolated body showed that targets’ perceived music preferences predicted their actual music preferences for energetic/rhythmic music in both poses; standardized poses revealed targets’ intense/rebellious music preferences and spontaneous poses marginally revealed targets’ reflective/complex music preferences; no other results reached significance. Excluding targets’ heads to show only their bodies thus eliminated music preference cues for reflective/complex music but not for intense/rebellious music or for energetic/rhythmic music. Indeed, these findings complement those of past studies underscoring the importance of face cues in valid social judgments (see Alaei & Rule, 2016). We therefore examined cues to music preference available in face and head cues.

#### Head and hair

Using models analogous to the full body photos, we observed that targets’ perceived music preferences based on their standardized and spontaneous head photos predicted their actual preferences for energetic/rhythmic, intense/rebellious, and reflective/complex music, although only marginally for energetic/rhythmic music in the standardized pose, and for intense/rebellious in the spontaneous pose. By contrast, targets’ perceived music preferences based on their hair photos did not predict their actual music preferences (Supplemental Table S8). Moreover, correlations between judgments based on targets’ hair and head show that hair only moderately contributed to judgments of the heads (all *r*s ≤ .40, all *p*s < .001), suggesting that the cues to targets’ music preferences likely reside within their faces.

#### Face

Statistical models paralleling those described above showed that targets’ perceived music preferences predicted their actual music preferences for energetic/rhythmic music in standardized photos, and for intense/rebellious and reflective/complex music in spontaneous photos (Supplemental Table S8). Thus, although the entire head better facilitated detection, these results suggest that some music preference cues persist in the face’s internal features (e.g., the eyes). The lower accuracy level suggests that some head judgments may have relied on cues not available in the internal face, such as those around the mouth (i.e., jaws and necks). We therefore tested independent contributions of the eye and mouth regions using analogous models.

#### Eyes and mouth

Perceptions of targets’ music preferences from each of their eyes and mouth predicted their actual music preferences for intense/rebellious music in the standardized poses, and just from their mouths in the spontaneous poses. Mouth cues in the spontaneous pose moreover revealed targets’ preferences for reflective/complex music. Multiple trait and behavioral cues surrounding the mouth may signal individuals’ music preferences, such as their attractiveness (e.g., facial blemishes, asymmetry), emotions (e.g., lips’ shape), and dominance and masculinity (e.g., neck width, jawline). We thus explored how trait and behavioral cues support judgments of music preferences in Study 2.

## Study 2: Subjective Cues

In Study 1, we found that people’s appearance conveys information about their music preferences. We reasoned that music preferences may be legible from photos because people’s music preferences reflect their social group memberships and individual traits, which are also evident in appearance (e.g., Rentfrow & Gosling, 2003).

Thus, traits inferred from targets’ appearance may allow for diagnostic judgments of their music preferences. To identify the apparent behavioral characteristics that may cue music preferences, we took a three-step approach. First, we reviewed the literature on personality trait correlates of music preferences, selecting those that most robustly associated with music preferences (i.e., most often significant and relatively large in magnitude; for example, Dollinger, 1993; Langmeyer et al., 2012). Second, we selected characteristics found to cue personality traits from full body photos (Naumann et al., 2009). Finally, we selected traits central to person perception (Nielsen & Kernaleguen, 1976; Sutherland et al., 2013). The final selection included the following appearance characteristics related to personality traits: style (distinctive and unique style vs. ordinariness), energetic, neatness (neat and tidy vs. messy), and relaxed (vs. tense), as well as attractiveness and dominance (dominant and powerful vs. submissive)—key aspects of appearance from person perception research, more broadly.

To examine the cues that signaled music preferences, we used the Lens model of accurate interpersonal judgments (Back & Nestler, 2016), a prominent model in social perception research also used to investigate correlates of personality traits in everyday life (e.g., Gosling et al., 2002). For people to accurately judge others, their stereotypes (i.e., *utilized cues*) must align with the *valid cues* that coincide with targets’ actual attributes. For instance, people stereotype fans of energetic/rhythmic music as looking dominant, fans of reflective/complex music as looking neat and distinctive, and fans of upbeat/conventional music as seeming submissive (DeYoung et al., 2013; Rentfrow & Gosling, 2007). Thus, if an individual looks dominant, they may be perceived to be a fan of energetic/rhythmic music (utilized cue). If looking dominant actually correlates with liking energetic/rhythmic music (valid cue), then accuracy will be achieved. This model therefore allowed us to investigate the validity of stereotypes about music fans.

### Method

We randomly assigned 600 MTurk Workers (333 women, 264 men, three undisclosed; *M*^age^ = 42.80 years, *SD* = 12.50; 73.67% White, 8.83% Black, 5.67% Hispanic/Latin, 5.17% East Asian, 2.17% Other, 1.83% South Asian) to judge a random subset of 21 of the same standardized targets from Study 1 as to whether they appeared attractive, dominant, powerful, submissive (reverse coded), distinctive in style, unique in style, ordinary in style (reverse coded), energetic, neat, tidy, messy (reverse coded), relaxed, and tense from 1 (*not at all*) to 7 (*a great deal*) in a between-subjects design that yielded an average of 43.60 (*SD* = 6.15) perceivers rating each target. Participants’ ratings showed high interrater reliability for each trait (see Supplemental Table S21 for Cronbach’s αs and Supplemental Table S22 for zero-order correlates).^6^ Because we used targets as the unit of analysis, statistical power mirrored that reported in Study 1.

Rather than ask participants to rate all stimulus versions from Study 1, we focused on the standard-pose full body photos because they had the highest ecological validity.

### Results and Discussion

We performed a CFA using jamovi version 1.8.1 (The jamovi project, 2021) with a robust maximum likelihood estimator (appropriate for the continuous consensus trait ratings) and listwise deletion for missing data to test a model that combined traits that were similarly worded (e.g., *messy appearance*, *tidy appearance*, and *neat appearance*) or conceptually related (e.g., *dominant* and *submissive*; Tiedens & Fragale, 2003). A model comprised of five correlated factors, including attractiveness, dominance (dominant and submissive), energetic, style (distinctive style, unique style, and ordinary style), and neatness (neat appearance, tidy appearance, and messy appearance),^7^ showed an adequate-to-good fit according to Hu and Bentler’s (1999) recommendations, χ^2^(25) = 72.08, *p* < .001, CFI = .99, RMSEA = .08, 90% CI = [.06, .10], Akaike information criterion (AIC) = 1,615.81, Bayesian information criterion (BIC) = 1,725.80; see Supplemental Figure S2 for the final model parameters. This model also included correlated residual errors for reverse-coded variables (distinctive style and messy appearance, distinctive style and submissiveness, and submissive and messy appearance) and equality constraints for the dominance factor (with two indicators) to help identify the model. A model comparison suggested that an oblique model with correlated factors showed a better fit, χ^2^(10) = 392.45, *p* < .001, than an orthogonal model with factor covariances fixed to 0, χ^2^(35) = 468.32, *p* < .001, CFI = .90, RMSEA = .21, 90% CI = [.19, .22], AIC = 2,217.36, BIC = 2,290.69.

This yielded five visual cues that predicted the targets’ actual and perceived music preferences in separate multivariate regressions, allowing estimation of both the valid and utilized appearance cues to individuals’ music preferences (see Table 2; see Supplemental Table S24 for results by target demographic group).

**Table 2.** Standardized Regression Coefficients Predicting Music Dimension Preferences From Perceived Traits Based on Full Body Photos in Study 2.

Music dimension								
Variables	Energetic/rhythmic		Intense/rebellious		Reflective/complex		Upbeat/conventional	
	Valid	Utilized	Valid	Utilized	Valid	Utilized	Valid	Utilized
Attractiveness	.15	.06	−.03	.06	.08	.05	−.04	.00
Dominance	−.04	.29***	.05	.19**	−.13†	−.18**	−.29**	−.19**
Energetic	.10	.27***	−.01	.08	.08	−.14†	.18*	.23***
Neatness	−.13†	−.43***	−.07	−.45***	−.20**	.37***	.02	.24***
Style	−.05	−.09†	.02	.01	.16**	.09	.00	−.15**
Control variables								
Age	−.02	−.23***	.02	−.24***	.19**	.27***	.08	.10*
Gender	−.10	−.16**	−.10	−.34***	−.09	.20**	.01	.56***
Race	−.08	−.18***	.10	.25***	−.04	.08	−.17**	.30***

*Note. N* = 289. Positive coefficients for Style represent more distinctive/unique style, whereas negative coefficients represent ordinary style. Coefficients for subjective cues derived from multivariate analyses statistically adjusting for age, gender, and race. Gender coded −1 = male, 1 = female. Race coded −1 = non-White, 1 = White.

† *p* < .10. *p < .05. **p < .01. ***p < .001.

#### Valid cues

Targets who preferred energetic/rhythmic music looked marginally less neat. Targets who preferred intense/rebellious music did not differ on any of the traits. Targets who preferred reflective/complex music appeared more submissive, distinctive in dress, messy, and were older. Finally, targets who preferred upbeat/conventional music appeared less dominant, marginally more energetic, and were more often non-White.

#### Utilized cues

The five cues and three social group memberships also predicted *perceptions* of targets’ music preferences. Alongside the valid cues correctly utilized, perceivers also utilized several invalid cues. Namely, they erroneously believed that people who looked relatively dominant, energetic, ordinary in style, younger, male, and non-White preferred energetic/rhythmic music; that people who looked relatively more dominant, messy, younger, male, and White preferred intense/rebellious music; that people who looked relatively neat, unenergetic, and female preferred reflective/complex music; and that people who looked relatively neat, ordinary in style, older, female, and White preferred upbeat/conventional music.

### Discussion

Study 2 suggests that people detect others’ preferences for energetic/rhythmic and upbeat/conventional music based on perceptions of their attributes. The valid cues in the analyses aligned with previously observed stereotypes (Rentfrow & Gosling, 2007): A messy appearance validly signaled a preference for energetic/rhythmic music, submissiveness validly signaled a preference for reflective/complex music, and submissiveness and energy validly signaled a preference for upbeat/conventional music. These stereotypes may be accurate because people prefer music that reflects their dispositions and regulates their energy levels (Sharman & Dingle, 2015; Sutoo & Akiyama, 2004).

Surprisingly, no perceived traits validly predicted preference for intense/rebellious music. Participants believed that young White men who appeared dominant, disheveled, younger, male, and White enjoyed intense/rebellious music. However, none of these utilized cues were valid.

Such stereotypes associating intense/rebellious music with excitement-seeking, delinquency, and antisocial traits may not be entirely accurate (Epstein et al., 1990). Rather, specific styles of dress (e.g., torn jeans, black shirts with heavy metal band logos, and leather jackets) and social group membership (White and male) may instead better predict preferences for intense/rebellious music (Epstein et al., 1990). Furthermore, people’s stereotypes of intense/rebellious music fans—such as having a dominant and disheveled appearance—may be outdated. Indeed, many intense/rebellious music genres became mainstream in the 1980s and early fans of bands such as *Metallica* and *Iron Maiden* are now part of the socially dominant, middle-aged workforce.

Consequently, the social ascension of these fans may have elevated these genres from being associated with a “low-brow” taste culture to now a “middle-brow” taste culture. Similar social promotions occurred for jazz and blues during the 20th century; music once stereotyped as “unruly” and “rebellious” may become normalized over time and thus part of mainstream culture (Fox & Wince, 1975; Lena & Peterson, 2008).

In terms of social category cues, younger and male targets were stereotyped as more likely to listen to energetic/rhythmic and intense/rebellious genres, whereas relatively older and female targets were stereotyped as more likely to listen to reflective/complex and upbeat/conventional genres. White targets were also stereotyped as more likely to enjoy intense/rebellious and upbeat/conventional music, whereas energetic/rhythmic music was believed to be preferred by non-White targets. These results coincide with previously reported stereotypes and valid differences in music preferences between demographic groups (e.g., preferences for rap and soul/funk with non-White groups, or classical music among older adults; Cohrdes et al., 2017). Finally, the findings also suggest that music dimension stereotypes align with the broader social dimensions of agency: Whereas stereotypes of reflective/complex and upbeat/conventional genres fans relate to stereotypically low dominance or submissive groups (such as older adults and women), stereotypes of energetic/rhythmic and intense/rebellious music fans related to highly agentic or dominant groups, such as young adults and men (Bakan, 1966; Sczesny et al., 2019). Such patterns suggest that fundamental interpersonal traits also inform stereotypes of music preferences and speak to the redundancy of these social traits across modalities.

Studies 1 and 2 collectively demonstrate that music preferences are identifiable from appearance by relying on perceptions of targets’ social group memberships and traits. In Study 3, we further examined the social importance of perceptions of others’ music preferences by investigating whether perceivers use these perceptions to guide their social intentions toward others.

## Study 3: Social Outcomes

If people “leak” their music preferences through their appearance, then others may use this information to inform social decisions. We therefore tested in Study 3 whether inferring music preference from appearance cues has downstream consequences. If music preferences function similarly to other types of social groups or traits, then people should express homophilous social preferences as a function of music preferences (Hehman et al., 2018; Lonsdale, 2020). For instance, people may be more interested in meeting individuals with similar music preferences and may also want to avoid individuals whose music preferences differ.

To test these hypotheses, we asked participants to judge how much they would like to meet individuals with varying music preferences. More specifically, we hypothesized that a match between the participants’ music preferences and the targets’ actual music preferences would influence how interested the participants would be in meeting the target. In addition, we tested whether a match between participants’ music preferences and the targets’ *perceived* music preferences would influence the participants’ interest in meeting the target. Participants’ perceptions of targets’ music preferences (regardless of validity) may better predict social intentions than targets’ actual music preferences because individuals act according to their beliefs.

### Method

Four-hundred MTurk workers (200 women, 197 men, three undisclosed; *M*_age_ = 41.64 years, *SD* = 12.72; 74.25% White, 10.00% Black, 5.50% East Asian, 4.75% Hispanic/Latin, 3.00% Other) evaluated how much they would like to meet a random subset of 95 targets from the pool of 289 on a scale from 1 (*not at all*) to 7 (*a lot*).^8^ We excluded one participant who completed the study twice. Similar to Studies 1 and 2, participants also completed the STOMP scale at the end of the target-rating block. Approximately 130.82 participants (*SD* = 9.44) rated each target. A power analysis suggested that the participant and target sample sizes provided over 99% power to find an effect size typical of interpersonal perception research (*r*_ES_ = .29; Tskhay & Rule, 2013) using a cross-classified multilevel model, assuming a 5% false-positive rate (Westfall et al., 2014).

### Results

We first examined whether the three-way Music Dimension × Target (Actual) Music Preferences × Perceiver (Actual) Music Preferences interaction predicted the perceiver’s desire to meet the target using a cross-classified model with random intercepts for perceivers (intraclass correlation coefficient [ICC] = .41) and targets (ICC = .11) and an unstructured covariance matrix to estimate all multilevel random effects.^9^ The model included mean-centered predictors and adjusted for targets’ age, race (effect-coded White = 1, non-White = −1), and gender (effect-coded female = 1, male = −1). Contrary to our hypothesis, the three-way interaction did not predict participants’ desire to meet the target, *F*(3, 151,522) = 0.96, *p* = .41, partial *R*^2^ < .001.^10^ Results also revealed no two-way interactions or simple main effects of experimental conditions, |β|s < .005, |*t*|s < 1.38, *p*s > .17, partial *R*^2^s < .001.^11^

Although an actual match between the perceivers’ and targets’ music preferences did not predict desire to meet, a match with the targets’ *perceived* music preferences did predict desire to meet in an analogous model (ICC_perceivers_ = .41; ICC_targets_ = .11), *F*(3, 151,522) = 4.51, *p* = .003, partial *R*^2^ < .001 (see Supplemental Figure S3). Follow-up analyses revealed that ostensible music preferences mattered more for some music dimensions than others (Table 3; see Supplemental Table S25 for results by target demographic group). For instance, a match for energetic/rhythmic music preferences predicted participants’ desire to meet the targets, whereas a match for intense/rebellious and reflective/complex music preferences did not. We also noted a marginal result for a match in upbeat/conventional music preferences. Last, we found that participants wanted to meet targets who appeared to enjoy upbeat/conventional music and reflective/complex music, regardless of the participant’s own preference for these music dimensions.

**Table 3.** Standardized Regression Coefficients for Matches Between Target’s Perceived and Perceivers’ Actual Music Preferences by Music Dimension.

Music dimension				
Variables	Energetic/rhythmic	Intense/rebellious	Reflective/complex	Upbeat/conventional
Target perceived preference (A)	−.002	−.02	.04*	.08***
Perceiver actual preference (B)	.19***	.16***	.18***	.16***
Interaction (A × B)	.01***	−.01	.003	.01†
Control variables				
Target age	−.07***	−.07***	−.07***	−.07***
Target gender	−.12***	−.11***	−.11***	−.07***
Target race	.06***	.07***	.06***	.04***

*Note.* Target perceived preference (A) represents how each target’s perceived preference for the music dimension predicts perceivers’ desire to meet the targets. Perceiver actual preference (B) represents how the perceiver’s preference for the music dimension predicts their desire to meet targets in general. Interaction (A × B) represents how a match between the perceiver’s actual preference and the target’s perceived preference for the music dimension predicts the perceiver’s desire to meet the target. Target *N* = 289, perceiver *N* = 399. Gender coded −1 = male, 1 = female. Race coded −1 = non-White, 1 = White.

† *p* < .10. *p < .05. **p < .01. ***p < .001.

### Discussion

These results suggest that people may use music preferences to guide their social intentions and behaviors. Contrary to our hypothesis, a match between the target’s and perceiver’s *actual* music preferences did not predict the perceiver’s desire to meet the target. Instead, matches between the targets’ *ostensible* preference predicted the perceivers’ desire to meet them in the case of energetic/rhythmic music and upbeat/conventional music (albeit marginally for the latter).

We also found main effects of the targets’ perceived preference for reflective/complex and upbeat/conventional music, that is, participants wanted to meet targets who seemed like they would enjoy these genres. These results were foreshadowed by Study 2, in which targets perceived to appreciate these types of music also looked neat and submissive. Moreover, regardless of the music dimensions, people who enjoyed music more (in general) expressed greater interest in meeting targets. Indeed, this aligns with work suggesting that people who appreciate music are more extraverted and open to new experiences (Dollinger, 1993).

Finally, participants also reported greater desire to meet targets who appeared younger, female, and White. These trends likely reflect more general positivity biases, such as halo effects for youthful faces, stereotypes of women as warm and communal, and same-race biases (Berry & McArthur, 1985; Eagly & Steffen, 1984; Fisman et al., 2008).

In sum, Study 3 suggests that music preferences may affect social outcomes for both targets and perceivers. Preferences emitted through stereotypical appearances may thus affect the social opportunities available to individuals.

## General Discussion

Individuals’ music preferences divulge much about them, as does their appearance (e.g., personality traits and values; Alaei & Rule, 2016; North & Hargreaves, 1999; Rentfrow & Gosling, 2007). We therefore investigated whether one’s appearance acts as a badge of their music preferences that affects their social lives. Across three studies, we found that people’s appearance revealed their music preferences, and that appearance cues signaling their music preferences affected social intentions toward them (see Table 4 for a summary of the findings).

**Table 4.** Summary of Physical Features and Psychological Attributes Diagnostic of Targets’ Music Preferences in Studies 1 to 3.

Music dimension					
Study	Variables	Energetic/rhythmic	Intense/rebellious	Reflective/complex	Upbeat/conventional
1	Photo condition	Full body, isolated body, head	Isolated body, head, internal face, mouth	Full body, head, mouth	Isolated body
2	Utilized cues	Dominance, high energy, messiness, ordinary style, younger age, male, non-White	Dominance, messiness, distinctive style, younger age, male, White	Submissiveness, low energy, neatness, older age, female	Submissiveness, high energy, neatness, ordinary style, older age, female, White
	Valid cues	Messiness	None	Submissiveness, messiness, distinctive	Submissiveness, high energy, non-White
	Accurate cues	Messiness	None	Style, older age submissiveness, older age	Submissiveness, high energy
3	Music dimension	Perceiver, match	Perceiver	Target, perceiver	Target, perceiver, match
	Preference → Desire to Meet				

*Note.* Photo condition summaries represent averaged results across standardized and spontaneous pose photos. “Target” represents how each target’s perceived preference for the music dimension relates to perceivers’ desire to meet the targets. “Perceiver” represents how the perceiver’s preference for the music dimension predicts their desire to meet targets in general. “Match” represents how a match between the perceiver’s actual preference and the target’s perceived preference for the music dimension predicts the perceiver’s desire to meet the target.

Specifically, preferences for energetic/rhythmic music were discernible from people’s full bodies, isolated bodies, heads, and faces; preferences for intense/rebellious music were visible from individuals’ isolated bodies, heads, faces, eyes, and mouths; and preferences for reflective/complex music were visible from individuals’ full bodies, heads, faces, and mouths. Preferences for upbeat/conventional music were not at all visible, however (possibly because fans lacked distinguishing cues or because participants in urban cities hesitated to express country/gospel music preferences). Moreover, many of these judgments were accurate for standardized neutral poses and for spontaneous idiosyncratic poses. Accurate perceptions of music preferences therefore seem robust to ephemeral variations in appearance.

In addition to being diagnostic, impressions of others’ music preferences also guide social intentions. Namely, the results suggested that perceivers prefer to meet targets who appear to match their preference for energetic/rhythmic music (e.g., perceivers who enjoyed energetic/rhythmic music preferred to meet targets who appear to enjoy energetic/rhythmic music over targets who did not appear to enjoy energetic/rhythmic music) and (marginally) for upbeat/conventional music. In addition, the more that individuals appeared to prefer reflective/complex and upbeat/conventional music, the more others preferred them as potential social partners, likely because they looked submissive, female, and White. Finally, people who enjoy music more in general (i.e., regardless of music dimension) express greater motivation to meet others, possibly reflecting music fans’ higher extraversion and openness to new experiences (Dollinger, 1993). Perceptions of others’ music preferences thus also influence social outcomes.

### Source of Music Preference Cues

Heads revealed more than bodies for intense/rebellious music and reflective/complex music, but just as much for energetic/rhythmic music. Thus, the head may convey more about one’s music preferences than the body does. This coincides with research showing that people use facial cues more often than body cues when forming first impressions, presumably because facial cues contain more valid information (Martinez et al., 2016). Buttressing this, we found that perceivers could discern music preferences better than chance even when further cropping the heads to individual features (i.e., the eyes and mouth). Because the participants serving as targets did not know the study’s purpose, music preferences seem incidentally encoded as deeply as these individual features. This may be unexpected, given that one might assume that purposeful, ephemeral cues such as dress or body movements (e.g., mannerisms) would determine music preferences’ legibility from appearance cues (Badaoui et al., 2012). The current findings instead suggest that stable facial features resistant to ephemeral alterations offer at least one prominent source of information about music preferences as well, dovetailing with literature suggesting that music preferences map onto people’s stable traits and values (Boer et al., 2011).

### Accuracy of Music Preference Stereotypes

These judgments do not rely solely on individuals’ age, race, or gender (although age, race, and gender did cue music preferences). Rather, apparent traits convey individuals’ music preferences over and above these basic dimensions (joining the associations between appearance-based traits and music preferences in previous research; for example, Penton-Voak et al., 2006; Rentfrow & Gosling, 2003), suggesting that cues to identity overlap across modalities. Specifically, people who prefer energetic/rhythmic music look messier, those who prefer reflective/complex music look submissive, and those who prefer upbeat/conventional music also look more submissive and energetic. These trait associations each correspond to stereotypes, thus suggesting “kernels of truth” (Rentfrow & Gosling, 2007). Indeed, the data show that people have strong stereotypes about fans of all genres (see Table 1). None of the idiosyncratic trait cues signaled individuals’ preferences for intense/rebellious music, however. Other relevant traits (e.g., impulsivity; Rentfrow, 2012) or perhaps cues more idiosyncratic to the genre (e.g., cosmetics) may reveal more.

Finally, participants could not identify upbeat/conventional music fans despite accurately stereotyping upbeat/conventional fans. One possibility is that upbeat/conventional music preferences may not be legible from appearance cues (Study 2) because invalid stereotypes hurt detection rates (i.e., more non-White than White targets preferred these targets, but people stereotyped White targets as more likely to enjoy upbeat/conventional music). A second possibility is that these genres lack obvious, visible subcultural music “scenes” with strong stereotypes and therefore have few distinctive intentional cues, by definition. Indeed, people (especially adolescents and young adults) use clothing and behaviors to transmit subcultural preferences to differentiate themselves from others (Frith, 1981; Zillmann & Gan, 1997). Thus, perhaps it was not surprising that preference for energetic/rhythmic, intense/rebellious, and reflective/complex music styles were discernible from isolated bodies because these types of music depart from mainstream culture. For instance, teenagers and young adults who listen to “problem” music, such as rap and hip hop, were negatively stereotyped as dangerous deviants in past decades (Mulder et al., 2007). By contrast, upbeat/conventional music preferences may not be apparent if people have outdated or no defined stereotypes about the music’s fanbase, which may also explain why participants did not recognize stable facial as valid indicators of preference for upbeat/conventional music.

### Social Outcomes of Inferred Music Preferences

Finally, the data suggest that perceptions of others’ music preferences affect social outcomes. Specifically, people who ostensibly prefer select music dimensions (i.e., reflective/complex and upbeat/conventional) look more approachable and thus elicit relatively higher intentions for social engagement. Moreover, we found that social outcomes depended on whether the perceiver believed the target shared their music preferences. Namely, people looked for cues signaling matching energetic/rhythmic preferences and upbeat/conventional preferences when determining whom they wanted to meet. At least for energetic/rhythmic music, this may be because fans of this music dimension seem like a more homogeneous social group than fans of other music dimensions. For example, rap and soul/funk genres (i.e., energetic/rhythmic music) have previously been associated with distinct racial minorities and low-socioeconomic groups (Tsitsos, 2018; see Table 2) and may therefore remain stereotyped as unconventional and low status, unlike alternative and rock music. As genres such as alternative and rock become more mainstream and less stigmatized (Earl, 2009), other genres necessarily become the new “badge of identity” for individuals at the fringes of society. The present findings thus provide the first evidence that appearance-based judgments engender different evaluations and reactions toward others based on their music preferences, perhaps also explaining why individuals favor others who share their music preferences (Lonsdale & North, 2009). People may therefore stereotype others based on perceived music preferences and regard individuals who appear to share their music preferences as ingroup members at first sight.

### Implications

Previous work on perceptions of ambiguous social groups has suggested that social identities are legible only when socially relevant to others (Rice & Mullen, 2003). Our findings may therefore suggest that music preferences constitute a relevant identity that people are broadly motivated to express and detect. But why? Similar to political ideology and religiosity (see Tskhay & Rule, 2013), music preferences may manifest in appearance because they encapsulate specific values and attitudes that help people to determine whom to approach and befriend (e.g., Boer et al., 2011; Gardikiotis & Baltzis, 2011). Music preferences might therefore serve as proxies for troves of information about people, efficiently accessed via appearance.

Importantly, the present findings provide additional insight into social identities that affect everyday social interactions and experiences otherwise neglected by psychological scientists (Rentfrow & Gosling, 2006). Indeed, such socially relevant attributes may help niche social groups to bond and maintain self-esteem but also become sources of discrimination as people look for clues of similarity and difference.

### Limitations and Future Directions

The current work has important limitations. First, we used one target set that may not equally represent all music preferences and we also assessed music dimension perceptions and appearance traits with only one sample of participants per component. Our target sample also included a limited range of racial and age groups, primarily consisting of East Asian and White young adults. As such, the distribution of music preferences among these target and perceiver cohorts restricted our ability to gauge the overall legibility and influence of music preferences. Indeed, the limited racial and age groups may account for the weak-to-moderate factor loadings for some genres (e.g., electronica/dance and rap/hip-hop) and inferior model fit indices when compared with the original STOMP model reported by Rentfrow and Gosling (2003; see Supplemental Figure S1). And although we offer an alternative music dimension model based on the 14 genres from the STOMP questionnaire (see Supplemental Materials), we also recognize that probing preferences for a different or greater selection of genres may yield new dimensions that better capture the music preferences of this target sample.

Similarly, we expect that other samples may show different patterns. For instance, although race did not validly cue energetic/rhythmic genres here, we recognize that this likely results from recruiting an East Asian majority sample that reported enjoying upbeat/conventional music the most. Race may validly cue energetic/rhythmic music preferences in predominately White and Black target samples (Marshall & Naumann, 2018). Similarly, a predominantly White target sample that matched the demographic composition of the participants would lead to accurate detection of upbeat/conventional music preferences because they would have savvier mental representations of the fans. Complementarily, preference for intense/rebellious music may prove indiscernible in regions where heavy metal music carries broad appeal (e.g., Finland; Grandoni, 2012). For this reason, we acknowledge that the current results do not definitively suggest that people *cannot* infer upbeat/conventional music preferences from appearance or that no valid cues exist for intense/rebellious music fans. Rather, the legibility of music preferences and other ambiguous social identities may depend on their eminence among targets and perceivers (Andrzejewski et al., 2009; Rule et al., 2010). This limitation echoes criticism that music-based subgroups only exist to the extent that people perceive and treat them as distinct subcultures (Zillmann & Gan, 1997). By recognizing the importance of music preferences to cultural and social movements (Eyerman, 2002; Futrell et al., 2006), future research may examine how impressions of targets’ (sub)culture (rather than just traits) underlie impressions of their music preferences or cultural tastes more broadly.

Future researchers might also pursue these possibilities because detection of intense/rebellious music preferences was among the most robust—apparent even from just the targets’ isolated eyes. Moreover, although we found that isolated features revealed music preferences, we did not examine whether people use all features equally when viewing the full body. Future work could therefore examine which features people attend when judging music preferences (e.g., via eyetracking). Relatedly, further exploration into the appearance cues to music preferences may help develop the theoretical mechanisms underlying the patterns observed here, a goal not best served by our data-driven approach emphasizing discovery.

This data-driven approach likewise involved multiple tests that could inflate the Type I error rate. We mitigated this risk via the family-wise error rates of multivariate regressions that reduce the number of independent tests (which several researchers have warned can lead to an inflated risk of Type II errors; for example, Althouse, 2016; Leary & Altmaier, 1980). Striking this balance proves difficult in exploratory work, yet the consistent and theoretically logical findings across the studies above suggest that the results are not spurious. Additional confirmatory testing would nevertheless help to further allay this concern.

Last, this work may lead to several directions concerning the consequences of interpersonal perception. Although we examined how perceived music preferences affect social outcomes, broadening to other preferences may also help to demonstrate how impressions of people’s preferences influence social evaluations. For instance, entertainment companies interested in algorithmically manufacturing music playlists for their consumers may even find that consumers’ appearance boosts their models’ ability to predict consumers’ preferences (e.g., by considering their user-profile pictures).

In sum, people’s music preferences are visible because they often reflect social identities and idiosyncrasies that are discernible from appearance cues. In turn, people use impressions of others’ music preferences to make social decisions. These results bolster previous work showing that music plays a significant role in people’s social lives (Rentfrow, 2012). Indeed, its significance begins with just a glance.

## Supplemental Material

sj-docx-1-psp-10.1177_01461672211048291 – Supplemental material for Appearance Reveals Music Preferences Supplemental material, sj-docx-1-psp-10.1177_01461672211048291 for Appearance Reveals Music Preferences by Laura Tian, Ravin Alaei and Nicholas O. Rule in Personality and Social Psychology Bulletin

## Notes

1. Although the comprehensive Short Test of Music Preferences–Revised (STOMP-R) Scale (Rentfrow, Goldberg, & Levitin, 2011) includes 23 genres instead of 14, we used the original scale to reduce participant fatigue (participants completed studies in addition to the one reported here); consulting the scales’ developer confirmed the original measure’s validity (P. J. Rentfrow, personal communication, May 9, 2018).

2. We removed five targets with headwear in the hair condition.

3. Participant samples were not equal because some participants completed the study after the MTurk HIT (Human Intelligence Task) expired.

4. We recruited approximately 40 perceivers per target in the hair condition because the hairstyles remained consistent between standardized and spontaneous poses.

5. Data and analysis scripts for all studies available at https://doi.org/10.17605/osf.io/uve6t

6. Despite their similarity to the Big Five traits, a literature search suggested that these traits more precisely align with music fan stereotypes than the generalized Big Five personality traits do (Schäfer & Mehlhorn, 2017).

7. A Vuong test (1989) comparing non-nested models suggested that a model utilizing all variables with an energy factor that included energetic, tense, and relaxed and a dominance factor that included dominant, submissive, and powerful showed a poorer fit than a model with energetic (alone) and a dominance factor consisting of dominant and submissive, *z* = 6.24, *p* < .001. A separate set of participants also rated the targets on positive demeanor and negative demeanor but a Vuong test showed that a model without Demeanor fit better than a model that also included a Demeanor factor (positive demeanor and negative demeanor), *z* = 21.86, *p* < .001.

8. Participants rated more targets than in Studies 1 and 2 to ensure that they would see a representative and diverse selection of targets from the pool.

9. Although participants and targets were crossed with music dimension, we did not include random intercepts for music dimension to avoid overfitting the model and because the random effect explained no variance, intraclass correlation coefficient (ICC) < .001.

10. Fractional degrees of freedom based on Satterthwaite approximations.

11. Results remained consistent when not accounting for either or both the targets’ and perceivers’ age, race, and gender.
