# Supplementary material for: Appearance Reveals Music Preferences
Source: Pers Soc Psychol Bull. 2021 Sep 28;48(12):1635–50. doi: 10.1177/01461672211048291 (PMC9606002; doi:10.1177/01461672211048291)
Supplement: sj-docx-1-psp-10.1177_01461672211048291 – Supplemental material for Appearance Reveals Music Preferences [file sj-docx-1-psp-10.1177_01461672211048291.docx]

**Stimulus Material**

**Study 1**

***Independent Variable: Targets’ Actual Music Preferences***

Instructions:

We are interested in your tastes in music. Please rate how much you like each genre from 1 (*Not at all*) to 7 (*A great deal*).

|  | Not at all  1 | 2 | 3 | 4 | 5 | 6 | A great deal  7 |
| --- | --- | --- | --- | --- | --- | --- | --- |
| Alternative |  |  |  |  |  |  |  |
| Blues |  |  |  |  |  |  |  |
| Classical |  |  |  |  |  |  |  |
| Country |  |  |  |  |  |  |  |
| Electronica/dance |  |  |  |  |  |  |  |
| Folk |  |  |  |  |  |  |  |
| Heavy metal |  |  |  |  |  |  |  |
| Rap/hip-hop |  |  |  |  |  |  |  |
| Jazz |  |  |  |  |  |  |  |
| Pop |  |  |  |  |  |  |  |
| Religious |  |  |  |  |  |  |  |
| Rock |  |  |  |  |  |  |  |
| Soul/funk |  |  |  |  |  |  |  |
| Sound tracks |  |  |  |  |  |  |  |

***Dependent Variable: Perceived Music Preferences***

Instructions:

You will be viewing a series of people’s photos and judging how much you think they like certain genres of music. You might experience that not every question is in view when you are looking at the picture. Please scroll down to look at the question and then scroll up to look at the picture before making each judgment.

Don’t spend too much time on any person or judgment; just go with your gut feeling! 
Note: Some pictures may take a few seconds to load. Please wait until the picture has loaded and close all other applications to ensure that the study functions properly.

You don’t need to answer the questions in this picture (the real experiment begins when you press next).

[Example photo and response scale here; see next page]

Press next to begin.

**Example Item**

[Target photo here]

|  | Not at all  1 | 2 | 3 | 4 | 5 | 6 | A great deal  7 |
| --- | --- | --- | --- | --- | --- | --- | --- |
| Alternative |  |  |  |  |  |  |  |
| Blues |  |  |  |  |  |  |  |
| Classical |  |  |  |  |  |  |  |
| Country |  |  |  |  |  |  |  |
| Electronica/dance |  |  |  |  |  |  |  |
| Folk |  |  |  |  |  |  |  |
| Heavy metal |  |  |  |  |  |  |  |
| Rap/hip-hop |  |  |  |  |  |  |  |
| Jazz |  |  |  |  |  |  |  |
| Pop |  |  |  |  |  |  |  |
| Religious |  |  |  |  |  |  |  |
| Rock |  |  |  |  |  |  |  |
| Soul/funk |  |  |  |  |  |  |  |
| Sound tracks |  |  |  |  |  |  |  |
| Happy music |  |  |  |  |  |  |  |
| Sad music |  |  |  |  |  |  |  |

**Study 3**

Instructions:

In this study, you will be viewing photos of people. Your task is to rate how much you want to meet them. You may not be interested in meeting any of them, but try to think about how interested you would be in meeting each individual if you had to.

Please use the scale provided, which ranges from **Not at all** (1) to **A lot**(7).

The task is not timed but you should work quickly. Just go with your "gut" instinct!
